# Case report: A unique quadruple coexisting anomaly—scimitar syndrome, atrial septal defect, vascular ring, and pulmonary sequestration

**DOI:** 10.3389/fped.2023.1214900

**Published:** 2023-07-18

**Authors:** Marcin Gładki, Paweł R. Bednarek, Wojciech Owecki

**Affiliations:** ^1^ Department of Pediatric Cardiac Surgery, Poznan University of Medical Sciences, Poznań, Poland; ^2^ Scientific Group of Pediatric Cardiac Surgery, Poznan University of Medical Sciences, Poznań, Poland

**Keywords:** pediatric cardiac surgery, scimitar syndrome, atrial septal defect, pulmonary sequestration, vascular ring

## Abstract

The article describes a successful clinical outcome in the case of a 5-month old female with a diagnosis of incomplete vascular ring of aberrant right subclavian artery and ostium secundum atrial septal defect associated with partial anomalous pulmonary venous return of scimitar syndrome type, coexisting with right pulmonary sequestration. During hospitalization, surgical correction of the heart defect and resection of the lung sequestration were performed. To the best of our knowledge, described constellation of defects is a unique phenomenon, posing a challenge for complex treatment and disease management.

## 1. Introduction

Scimitar syndrome is an uncommon congenital disease characterized by abnormal systemic arterial supply to the lower part of the right lung and, therefore, its underdevelopment, dextroposition, and total or partial anomalous pulmonary venous return (PAPVR) to the inferior vena cava (IVC) or right atrium. The aforementioned abnormal venous drainage produces a unique radiographic appearance which resembles a Turkish sword—the scimitar. Incidence of scimitar syndrome is low, it is estimated at about 1–3 per 100 000 live births, with female predominance reported (1, 2). If PAPVR involves the right upper pulmonary vein, it can be linked with a sinus venosus atrial septal defect (3). Clinical presentation of scimitar syndrome differs, it ranges from severe congestive heart failure occurring in infants to mild symptoms occurring in childhood (2).

Coexisting bronchial or tracheal anomalies in the form of sequestration, stenosis, or diverticulums have been reported (1). Pulmonary sequestration (PS) is also a congenital disease, defined by improper functioning of the lung tissue with anomalous connection to the bronchial tree and anomalous arterial systemic supply (4). As for the vascular ring, it is a congenital abnormality of the aortic arch that leads to esophageal or tracheal compression. Four categories of vascular rings were described: pulmonary artery sling, innominate artery compression, double aortic arch, and right aortic arch (RAA) with left ligamentum (5). Vascular rings were also divided into two categories: incomplete and complete (6). Lastly, atrial septal defect (ASD) is a congenital cardiac defect, which occurs when the interatrial septal tissue is absent or not sufficient. Three major types of ASD are distinguished: ostium primum, ostium secundum, and sinus venosus (7). Here we report coexisting anomalies of vascular ring, scimitar syndrome, intralobar PS, and ASD in an infant, which, to the best of our knowledge, is the first described case of such a quadruple anomaly.

## 2. Case description

A 5-month old female patient in severe condition presented to the Department of Pediatric Cardiac Surgery was diagnosed with scimitar syndrome, ASD, and right intralobar PS. Patient's symptoms at the presentation included respiratory distress and tachypnea. According to anamnesis, after birth as an infant she required continuous positive airway pressure (CPAP) therapy support for 3 days, followed by high-flow nasal cannula (HFNC) oxygen therapy due to respiratory failure. Postnatal genetic consultation included a karyotype analysis obtaining a normal test result. There was no available information on prenatal diagnosis.

**Figure 1:**
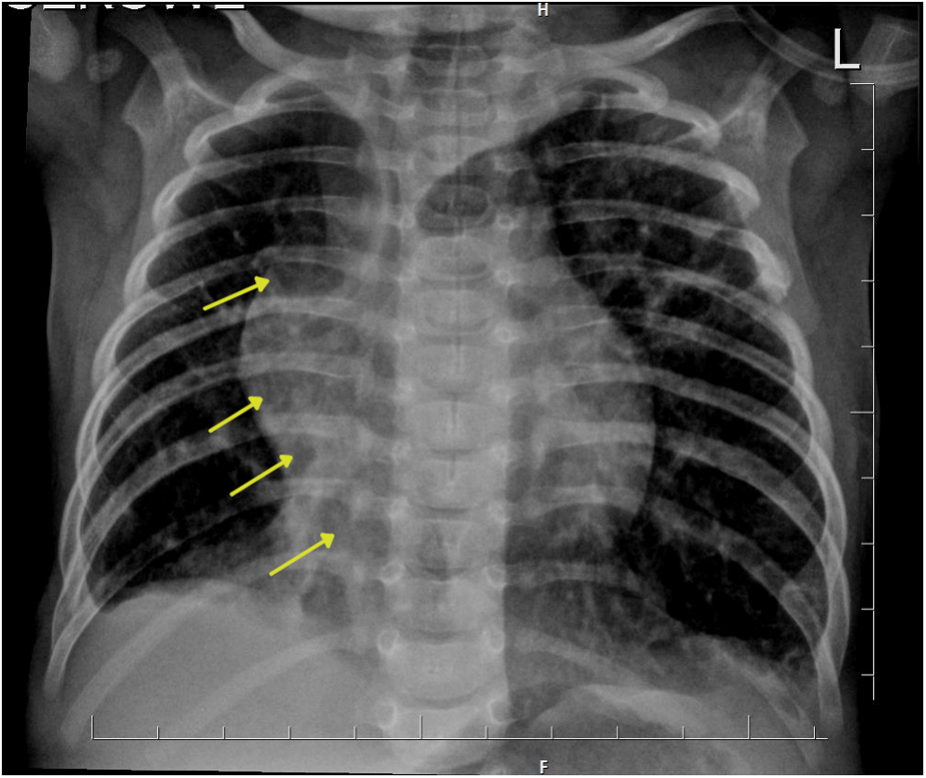
Chest x-ray presents the characteristic appearance of an anomalous vein resembling a Turkish sword, which is a typical image of scimitar syndrome. Arrows show this vein forming the common collector, which ends in the inferior vena cava.

**Figure 2:**
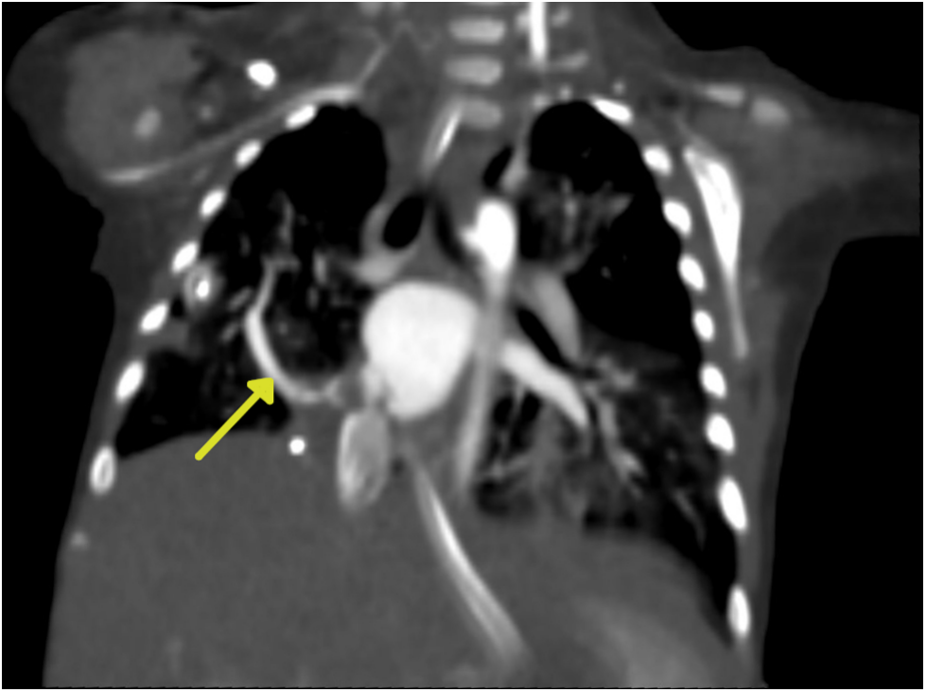
Computed tomography (CT) angiography. The yellow arrow indicates the arch-shaped silhouette of the scimitar vein.

**Figure 3:**
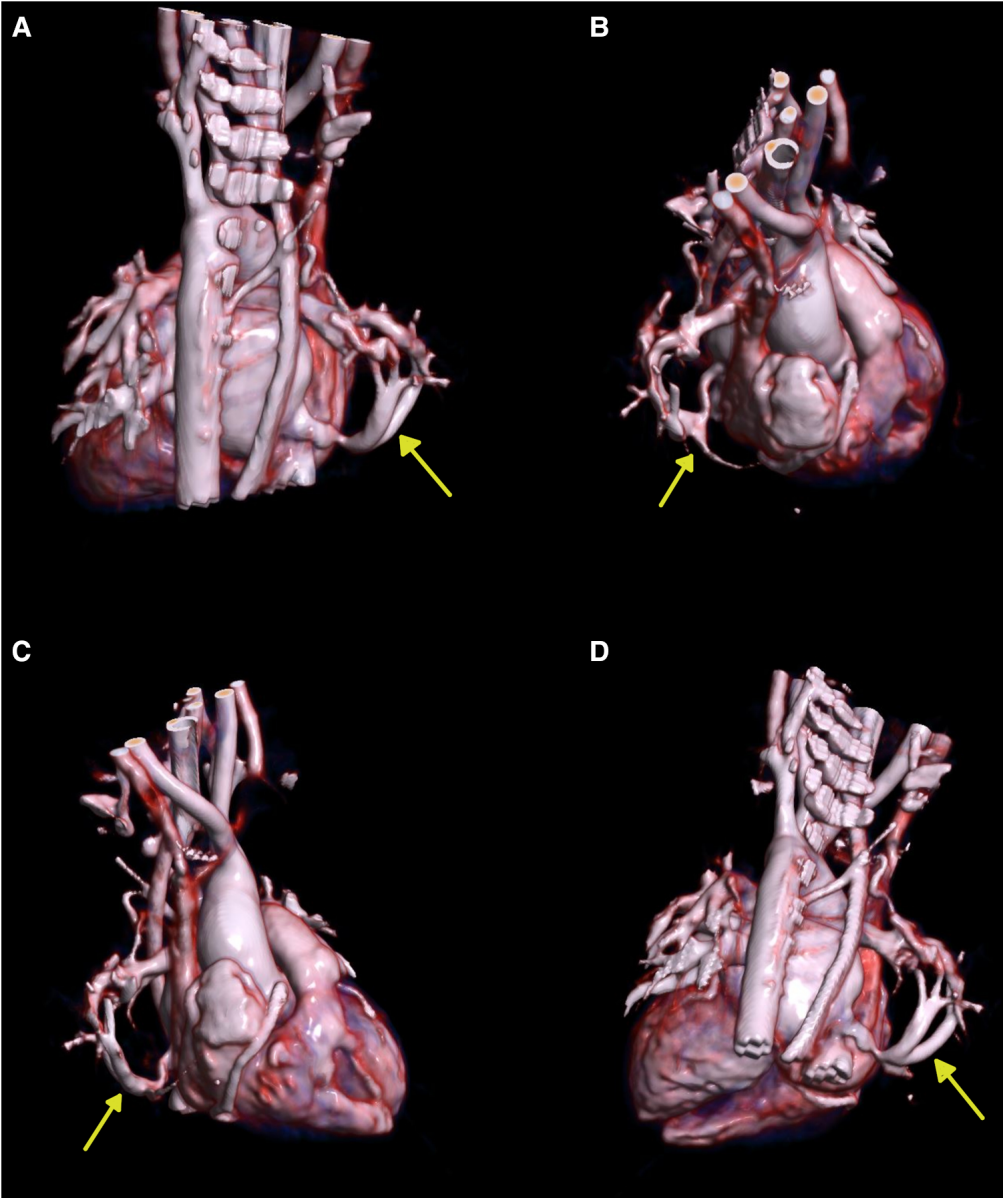
Three-dimensional (3D) volume-rendering reconstruction. (**A**) Posterior view. (**B**) Right superolateral view. (**C**) Right lateral view. (**D**) Posteroinferior view. The yellow arrows show the scimitar vein.

**Figure 4:**
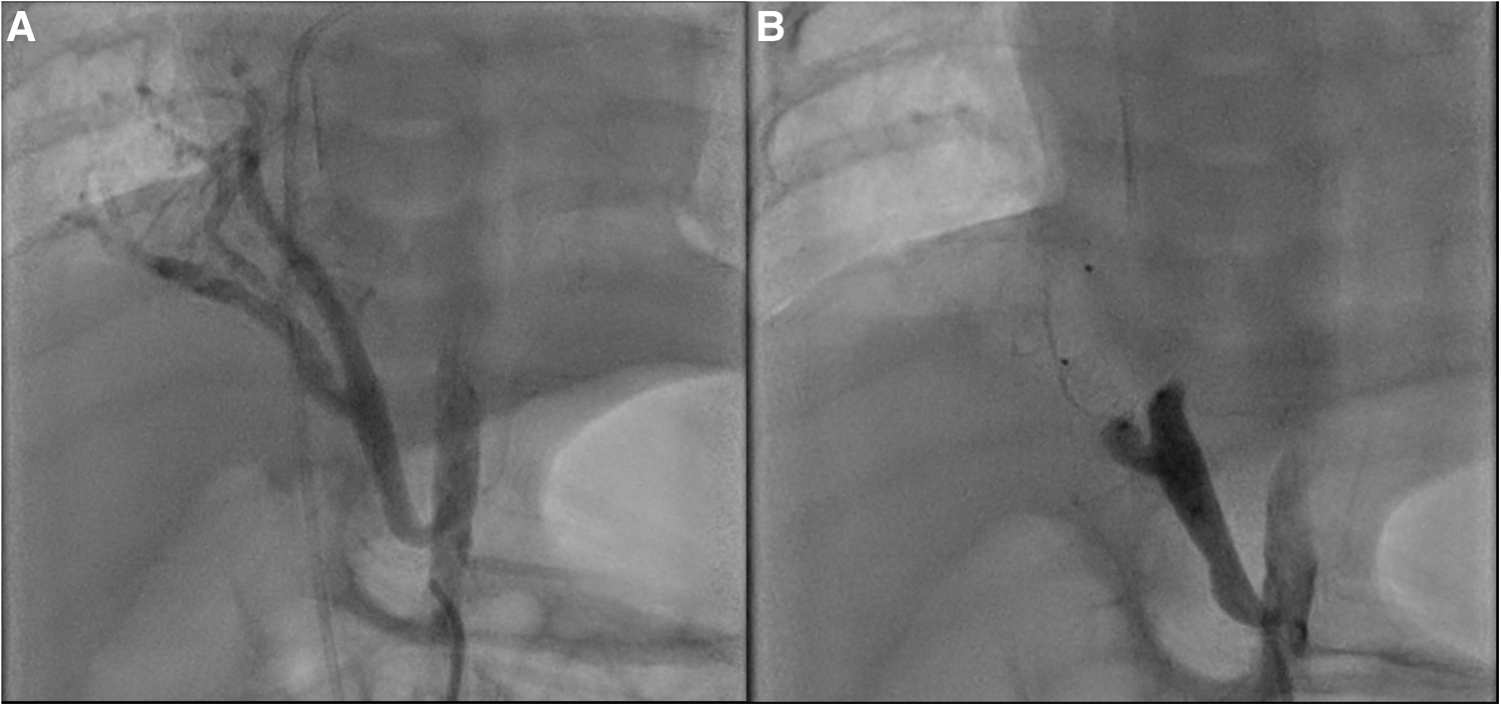
Angiography of the arteries supplying the right lung sequestrum from the aorta. (**A**) The artery heading upward and supplying part of the lower lobe was visualized. A short trunk about 4.5 mm in diameter, dividing into two arteries about 3.6–4.0 mm in diameter. (**B**) Embolization of both branches of the artery using Amplatzer Piccolo™ Occluder implants.

Chest x-ray showed the characteristic appearance of an anomalous vein in the shape of a Turkish sword, a typical radiographic feature for scimitar syndrome (Figure 1). Diagnosis of ASD associated with scimitar syndrome was based on transthoracic echocardiography, with the latter one subsequently confirmed by computed tomography (CT) angiography (Figure 2) and three-dimensional (3D) volume-rendering reconstruction (Figure 3). Earlier, at the age of 1.5 months, she underwent surgical correction of an incomplete vascular ring consisting of the aberrant right subclavian artery (ARSA, arteria lusoria). Then, at the age of 4.5 months, in a previously performed cardiac catheterization, angiography revealed major aortopulmonary collateral arteries (MAPCA) supplying pulmonary sequestration and PAPVR of the right lung draining through an arch-shaped common collector into the IVC, just below the level of the hepatic veins outlet (with normal pulmonary drainage from the left lung). During the procedure, both branches supplying the right PS were embolized with implantation of the Amplatzer Piccolo™ Occluder (Abbott) (Figure 4). All described procedures were performed in our hospital.

After admission, the patient was qualified for surgical correction of ASD. The pleural cavities and pericardium were opened from median resternotomy access. After cannulation of the aorta and both vena cavae, extracorporeal circulation was started. The aorta was cross-clamped, and blood del Nido cardioplegia solution was administered. The right pulmonary vein collector was dissected up to its outlet to the IVC. Visual *in vivo* assessment did not reveal any stenosis along the course of the veins. The right atriotomy approach exposed an overgrown Eustachian valve and ASD II. A collector was cut off from the IVC, and its end was replanted to the side of the incised free wall of the left atrium. The created anastomosis was then widened with a PhotoFix® patch, achieving an unobstructed blood flow. The Eustachian valve was subsequently removed, and the interatrial communication was closed with a patch. After de-airing of the left atrium, the aorta was declamped at 67 min, and the right atrium was closed. In the right pleural cavity, a 2-mm MAPCA vessel was located and ligated using a titanium clip. Extracorporeal circulation was gradually reduced and stopped at 127 min without complications. Hemostasis of the surgical field was achieved, but due to the child's preoperative condition, closure of the sternum was postponed, leaving an additional two pleural drains. Resection of the pulmonary sequestration was scheduled in the next stage of treatment to reduce the risk of systemic inflammatory response syndrome (SIRS) due to prolonged surgery.

In the postoperative period, the patient was mechanically ventilated in SIMV mode (FiO_2_ 60%) and circulatory supported with catecholamines. X-ray revealed increased density of middle and lower parts of the right lung parenchyma and hilar part of the left lung parenchyma. Moreover, blurred heart and right diaphragmatic dome silhouettes were observed. On the 2nd day after surgery, the chest was revisioned and the sternum was closed. On the 10th day, pneumothorax appeared, in consequence drainage was applied. Subsequently, the echocardiographic examination was performed with obtained parameters as follows: LV: 1.6/0.7 cm, RV: 1.0 cm, LVEF: 88%, MFV in both ascending and descending aorta was 1.0 m/s. The patient became feverish during hospitalization—after microbiologic diagnosis, proper antibiotic therapy was applied. A surgical consultation based on CT scan qualified the patient for her third operation—surgical resection of the intralobar PS, which was performed 27 days after surgical correction of ASD and scimitar syndrome.

The procedure was performed from a right-sided thoracotomy access through the 7th intercostal space. In the surgical field, multiple adhesions of the lower lobe of the right lung with the mural pleura and the dome of the diaphragm were visualized and subsequently released. The erosion of the diaphragmatic dome was then supplied with single sutures. A dark blue area of ischemically altered sequestration in the posterior-basal part of the lower lobe of the right lung was identified as intralobar sequestration. Three arteries penetrating through the diaphragm into the lower lobe of the lung were located: a medial vessel occluded with an external clip, a middle vessel poorly filled but still with a palpable pulse, and a lateral vessel occluded with an intraluminal coil. After clipping and dissecting all three arteries, the ischemically altered segment of lung parenchyma was resected using an ECHELON™ endoscopic stapler. Furthermore, a longitudinal section of the lower lobe of the lingula-morphology lung was also removed due to lack of aeration. A suction drain was left in the pleural cavity, and the intercostal, muscle, and skin tissues were sutured in layers.

After the procedure, the patient was mechanically ventilated in SIMV mode (FiO_2_ 35%), sedated, and supported with catecholamines. Control chest x-ray revealed increased density of the right lung parenchyma (as before) and liquid in the right pleural cavity. Later, on the 16th day after surgical resection of PS, a temporary tracheotomy was performed and SIMV ventilation was gradually reduced. The tracheostomy tube was removed 19 days later, she could breathe effectively by herself. Sedation was reduced, and with time the overall condition of the patient improved.

Due to an enlarged liver and persistent postprandial vomiting, on the 54th day after resection of PS, the patient was transferred to the Department of Pediatric Gastroenterology for further diagnostics and treatment due to intolerance to attempts of oral nutrition. Percutaneous endoscopic gastrostomy (PEG) feeding was implemented. On 79th day, the patient was discharged from the hospital in good general condition.

At 10 months of age, the patient has undergone a follow-up cardiovascular assessment. Follow-up echocardiography showed no significant deviations, except for small tricuspid regurgitation.

## 3. Discussion

To the best of our knowledge, this is the first report regarding such a quadruple anomaly. In the past, Jain and others reported a case with the scimitar syndrome, vascular ring (in the form of ARSA), and gastrointestinal bleeding, however, PS was not mentioned. Moreover, that case regarded a 57-year old woman with an aberrant right subclavian artery–esophageal fistula, which developed following surgical correction of scimitar syndrome. The latter procedure was performed with a use of a 20-mm dacron graft anastomosed to the orifice of the pulmonary veins, subsequently connected through the atrial septostomy with the right atrium. In that case, open tracheostomy was performed on the 8th postoperative day (8). Scimitar syndrome coexisting with PS was described in several case reports (9–11). In addition, a dual anomaly consisting of vascular ring (in the form of double aortic arch) and bronchopulmonary sequestration was reported (12). The presence of ASD II makes our case even more unique. In the past, scholars reported cases regarding the presence of ASD associated with each of the remaining three aforementioned anomalies. Juraszek and others reported ASD ostium secundum coexisting with the scimitar syndrome. In that case, the management of these anomalies was separated. During surgical treatment of scimitar syndrome, the left pulmonary venous confluence was corrected with a side-to-side anastomosis to the left atrium. This procedure included ligation and division of the distal part of the abnormal venous connection. The patient recovered without complications (13). Ammash and others in their study reported that among patients of all ages, presented with PAPVR, sinus venosus ASD was the most common associated congenital heart anomaly (49% of cases), while ASD II was much less common (only 13.3%) (14). ASD presented with intralobar PS was described by Itoh and others (15). Lastly, Ohye and others reported ASD associated with vascular ring (16). In yet another study, Youssef and others described a case of a 9-year old female diagnosed with scimitar syndrome, ARSA, and ASD II. They also reported coexistence of urinary anomalies, bronchus obstruction, and diaphragmatic hernia. In that situation, the management of scimitar syndrome was performed through mobilization of the pulmonary venous collector into the pericardial cavity. The aforementioned collector was incised, then an incision was made on the left atrium wall as well, and both structures were connected with sutures. ASD was closed with a pericardial bovine patch, and the pulmonary collector was reversed to the left atrium. The Eustachian valve was partially resected. The patient's recovery was positive (17). In a different paper, Noble and others reported a case of coexisting ASD II, PS, and scimitar syndrome, however, that study regarded a 57-year old woman. Considering her age, the Authors decided on a stepwise transcatheter method using Amplatzer devices. The transcatheter ASD closure with transesophageal echocardiography guidance was performed, which was preceded by balloon sizing of the defect. Subsequently, the abnormal arterial vessels to the lung sequestration were embolized. The whole procedure took 114 min (18).

ASD is the second most common congenital heart anomaly, with incidence estimated at 1.65 per 1,000 live births (19) and female predominance reported. ASD ostium secundum, which was diagnosed and treated in this case, is located by fossa ovalis, and usually appears because of a defect or several defects within septum primum. During childhood, the majority of patients do not report symptoms (20). As for adults, the significance of clinical consequences depends on the size of ASD (21). Transthoracic echocardiography is believed to be the primary diagnostic method and is used for determining the presence, size, location, and hemodynamic parameters of ASD (20). Treatment of ASD depends on the age of the patient, degree of hemodynamic shunt, and existence of symptoms of congestive heart failure (22). Recommendation for ASD closure constitutes any hemodynamically remarkable shunt that causes significant right heart enlargement. Management includes surgical or percutaneous closure. Traditional median sternotomy approach and central cannulation is known to be a simple and safe method (23). Interestingly, ASD may close spontaneously before the age of 2 years (19).

The diagnosis of scimitar syndrome is not difficult if the chest x-ray clearly shows the abnormal vein. However, if the image is not unequivocal, different diagnostic modalities can be applied (24). Echocardiography is often used, nevertheless, it is not perfect because of its inability to create 3-dimensional images. Magnetic resonance angiography and computed tomography are being increasingly used, with computed tomography angiography considered as a modality of choice (25). Surgical treatment of scimitar syndrome including timing, indications, and type of intervention is not clearly specified. Multiple techniques have been reported. Chowdhury and others, in their study, reviewed 22 techniques. They mentioned division of the abnormal vein and its reimplantation into the right atrium, or translocation into the left or right atrium. Moreover, intra-atrial baffling, the IVC partitioning with rechanneling of the anomalous vessel into the left atrium, and scimitar vein cut back technique were described. In addition, the pericardial tunnel technique, *in situ* pericardial roll method, and modified *in situ* pericardial rerouting with repositioning of the atrial septum were also presented. Lastly, several more surgical approaches such as pneumonectomy, transcatheter therapy, repair by a novel multipatch technique, and other methods were described in their paper (1). In a recent study, Wang and others, in their retrospective analysis, reported the use of scimitar vein reimplantation and intra-atrial baffle methods. The choice of method depended on the pathological and anatomic features, as well as on surgeon preference. Out of seventeen patients surgically treated, the intra-atrial baffle was applied in seven cases, and in eight cases the scimitar vein was reimplanted (26). On the other hand, a different paper regarding 26 patients surgically treated, presents the dominance of intra-atrial baffle repair (eighteen patients) in contrast to scimitar vein reimplantation (eight patients) (27). Best surgical approach is not clearly defined (26, 27). The principle of surgery is to create an unlimited pathway for the scimitar vein through newly created or already existing interatrial communication without causing complications (1). In infants, medical intervention is indicated, however, if pulmonary hypertension develops, the intervention should be prompt (28). Interestingly, Wang and others compared expeditious surgical repair and conservative treatment of symptomatic infantile patients with scimitar syndrome. The surgery was associated with better 1-year survival (29). This remains consistent with a recent study, which suggests that aforementioned patients should undergo early surgical correction. Nevertheless, the preference of early or delayed surgical treatment in infants lacks accurate specification and demands further study (27). Despite that, generally, in all symptomatic patients surgical correction is recommended. However, as for asymptomatic patients, the surgery is advised when their pulmonary-to-systemic flow ratio is greater than 1.5:1 or lesser than this value in patients with present clinically treated pulmonary hypertension, concomitant heart lesions, or scimitar vein stenosis (1, 30). In adults, indication for surgical correction remains debatable. Nevertheless, generally approved schemes are similar to these applied for infants (1). It has been reported that there is an association between patients's age and the success of surgery. Patients, who require surgical intervention before 1 year old, are usually very ill. Moreover, their operative mortality and complication probability is relatively high. Conversely, older patients' results are better both immediately and after a longer period of time (31).

ARSA is a relatively common vascular anomaly with a prevalence estimated at 0.5%–1% of the world's population (5) and female predominance reported. This disorder is usually asymptomatic and found incidentally. ARSA creates an incomplete vascular ring usually by positioning posteriorly to the esophagus towards the right axilla (80% of cases), between the trachea and esophagus (15% of cases), and anteriorly to the trachea (only 5% of cases) (8). ARSA can result in esophageal compression causing dysphagia lusoria, recurrent laryngeal nerve compression causing Ortner syndrome, and tracheal compression, which results in dyspnea (32). From embryological point of view, this anomaly develops when the right fourth aortic arch abnormally curls spirally towards the cranium, to the seventh intersegmental artery. Treatment of ARSA must be suited individually to every patient. In pediatric patients, the most favored method is division of the vascular ring without reconstruction of the aforementioned artery. Conversely, this approach is not recommended in adults because non-revascularization of the excluded artery may lead to upper extremity ischemia or subclavian steal syndrome. The best management for adult patients is graft replacement of diseased or dilated vessels under cardiopulmonary bypass. Apart from open surgery methods, endovascular methods such as thoracic endovascular aortic repair (TEVAR) and hybrid techniques are also used in ARSA treatment (33).

PS is a rare congenital disease, which prevalence is believed to be about 0.225%–0.425% (34), and which is considered to be the second most common congenital lung anomaly. It is divided into two categories based on its pleural covering: intralobar sequestration (ILS) and extralobar sequestration (ELS) (35). ILS, which accounts for 75% of cases, is described as lung parenchyma that shares the exact visceral pleural lining of the adjacent lung, and has venous connection with pulmonary veins. In contrast, if the lung parenchyma has its own separate visceral pleural covering outside of the adjacent normal lung and has venous drainage into systemic veins, then it is ELS, which makes up for 25% of cases. ELS forms an accessory lobe, named “Rokitansky lobe” (35, 36). Treatment of PS involves surgical resection, including separation and division of abnormal systemic feeding arteries. This method is known to be the safest and most effective (37). It is recommended because of the low probability of recurrent infection, risk of hemorrhage, and development of malignancy. Usually, lobectomy is performed, however, wedge resection can also be done, especially for small lesions located further from the pulmonary hilum, surrounded by a remarkable amount of normal lung tissue. The surgical management can be performed via video-assisted thoracoscopic surgery (VATS) or open thoracotomy (36, 38). VATS was reported to be superior to open thoracotomy with regard to hospital stay, postoperative pain, and postoperative outcome. Preoperative endovascular embolization of large aberrant vessels is believed to be reasonable to prevent intraoperative bleeding, however, it may also cause complications (38). In contrast, when the anatomy is unclear and severe adhesions in the lower lung ligaments are present, the lobectomy can be performed first, before treatment of abnormal vessels (39).

In conclusion, such a complicated coexisting anomaly, as we describe, is a unique phenomenon. Treatment of patient with this disorder is very complex. It constitutes a high challenge, especially considering the fact that several complications may appear. Therefore, it is recommended that a team of highly-experienced specialists should be engaged to achieve successful management of such abnormality.

## Data Availability

The original contributions presented in the study are included in the article, further inquiries can be directed to the corresponding author.
